# Significance low oscillating magnetic field and Hall current in the nano-ferrofluid flow past a rotating stretchable disk

**DOI:** 10.1038/s41598-021-02633-0

**Published:** 2021-12-01

**Authors:** Muhammad Ramzan, Saima Riasat, Yan Zhang, Kottakkaran Sooppy Nisar, Irfan Anjum Badruddin, N. Ameer Ahammad, Hassan Ali S. Ghazwani

**Affiliations:** 1 Department of Computer Science, Bahria University, Islamabad, 44000 Pakistan; 2 School of Science, Beijing University of Civil Engineering and Architecture, Beijing, 100044 China; 3 Beijing Key Laboratory of Functional Materials for Building Structure and Environment Remediation, Beijing University of Civil Engineering and Architecture, Beijing, 100044 China; 4 Department of Mathematics, College of Arts and Sciences, Prince Sattam Bin Abdulaziz University, Wadi Aldawaser, 11991 Saudi Arabia; 5 Research Center for Advanced Materials Science (RCAMS), King Khalid University, P.O. Box 9004, Abha, 61413 Asir Saudi Arabia; 6 Mechanical Engineering Department, College of Engineering, King Khalid University, Abha, 61421 Saudi Arabia; 7 Department of Mathematics, Faculty of Science, University of Tabuk, Tabuk, 71491 Saudi Arabia; 8 Department of Mechanical Engineering, Faculty of Engineering, Jazan University, Jazan, 45124 Saudi Arabia

**Keywords:** Mechanical engineering, Mathematics and computing, Software

## Abstract

The present investigation involves the Hall current effects past a low oscillating stretchable rotating disk with Joule heating and the viscous dissipation impacts on a Ferro-nanofluid flow. The entropy generation analysis is carried out to study the impact of rotational viscosity by applying a low oscillating magnetic field. The model gives the continuity, momentum, temperature, magnetization, and rotational partial differential equations. These equations are transformed into the ODEs and solved by using bvp4c MATLAB. The graphical representation of arising parameters such as effective magnetization and nanoparticle concentration on thermal profile, velocity profile, and rate of disorder along with Bejan number is presented. Drag force and the heat transfer rate are given in the tabular form. It is comprehended that for increasing nanoparticle volume fraction and magnetization parameter, the radial, and tangential velocity reduce while thermal profile surges. The comparison of present results for radial and axial velocity profiles with the existing literature shows approximately the same results.

## Introduction

Magnetic particles of ferromagnetic materials are strongly magnetized subject to an external magnetic field. This novel property of magnetic particles leads to a new research field known as ferrohydrodynamic^1^. Rosensweig^2^ studied the concept of electromagnetism, fluid dynamics, and thermodynamics to understand the dynamics of magnetic fluids. Ferroliquid is a colloidal suspension of nanosized particles of iron. Ferroliquid consists of nanoparticles with a diameter (3–15 nm) per cubic meter. Vast industrial applications motivated the researcher to explore the magnetic properties of Ferro liquid. Verma and Ram^3^ investigated magnetic liquids by considering the mathematical model in the tensor form. It is concluded here that the flow rate is greatly affected by the curvature. Rinaldi et al.^4^ reviewed the development in the rheology of magnetic fluid. They extended the work by considering the ferrohydrodynamic equation with viscous stress tensor and magnetization relaxation parameters. The magnetic fluid in the presence of magnetic dipole moment to study the impact of various pertinent parameters. Moreover, both MHD and FHD effects were simultaneously considered in a numerical study^5–15^. A theoretical study of nanofluids with EMHD effects is addressed^16–19^. The impact of ferromagnetic interaction parameters on heat transfer is analyzed by various researchers^20–25^. When a magnetic field is applied two situations arise in the flow field. If the direction of vorticity and particle’s magnetic moment are collinear then the direction of magnetic field aligns in the direction of particle’s magnetic moment and hence no change occurs in viscosity. As a result, resistance increases, and the liquid gets the finer viscosity with additional dissipation appears as the rotational viscosity^26–29^. Vaidyanathan et al.^30^ investigated Ferro convective instability with the impact of magneto-viscous effect for a rotating system. Hence this magnetic field-dependent viscosity induces convection. Linear stability analysis of magneto-viscous fluid with rotation also become part of a study by various researchers^31,32^. Ram et al.^33^ give a theoretical examination of the magneto-viscous effect on a nanoferrofluid for rotating disk. Moreover, they found that in the case of non-collinear vorticity vector and applied magnetic field, the velocity components exhibit additional resistive forces due to effective magnetization parameters.

Heat exchangers, convective heat transmission by solar radiation, heat transfer around fins, as well as other practical applications are governed by the Newtonian heating process. Viscous dissipative nanofluid flow with Newtonian heating was examined by Makinde^34^. He concluded with the result that increasing Newtonian heating causes the thermal boundary layer thickness to increase. Sarif et al.^35^ reported Newtonian heating on unsteady MHD flow past a stretching sheet. The Newtonian heating effect was explored by Ramzan et al.^36^.

Entropy generation with Newtonian heating in Hydromagnetic flow due to radial stretching sheet was examined by Das et al.^37^. It is concluded that the strongest source of entropy is the surface of the sheet. Entropy generation investigation along with the impact of magnetic interaction parameters over rotating disk has been the key of interest for various researchers^38–41^.

The studies mentioned above unveiled that there are studies that discuss the flow of the nano ferrofluid flow in various geometries. However, this channel is narrowed down if the flow of the nano ferrofluid flow over a stretching rotating disk with the Hall current and low oscillating magnetic field. Impact of Joule heating, viscous dissipation, with entropy generation analysis and convective boundary condition enhance the novelty of the problem. Here, the effects of rotational viscosity on temperature and velocity profile are elaborated. The envisioned mathematical model is solved numerically.

## Problem formulation

**Figure 1:**
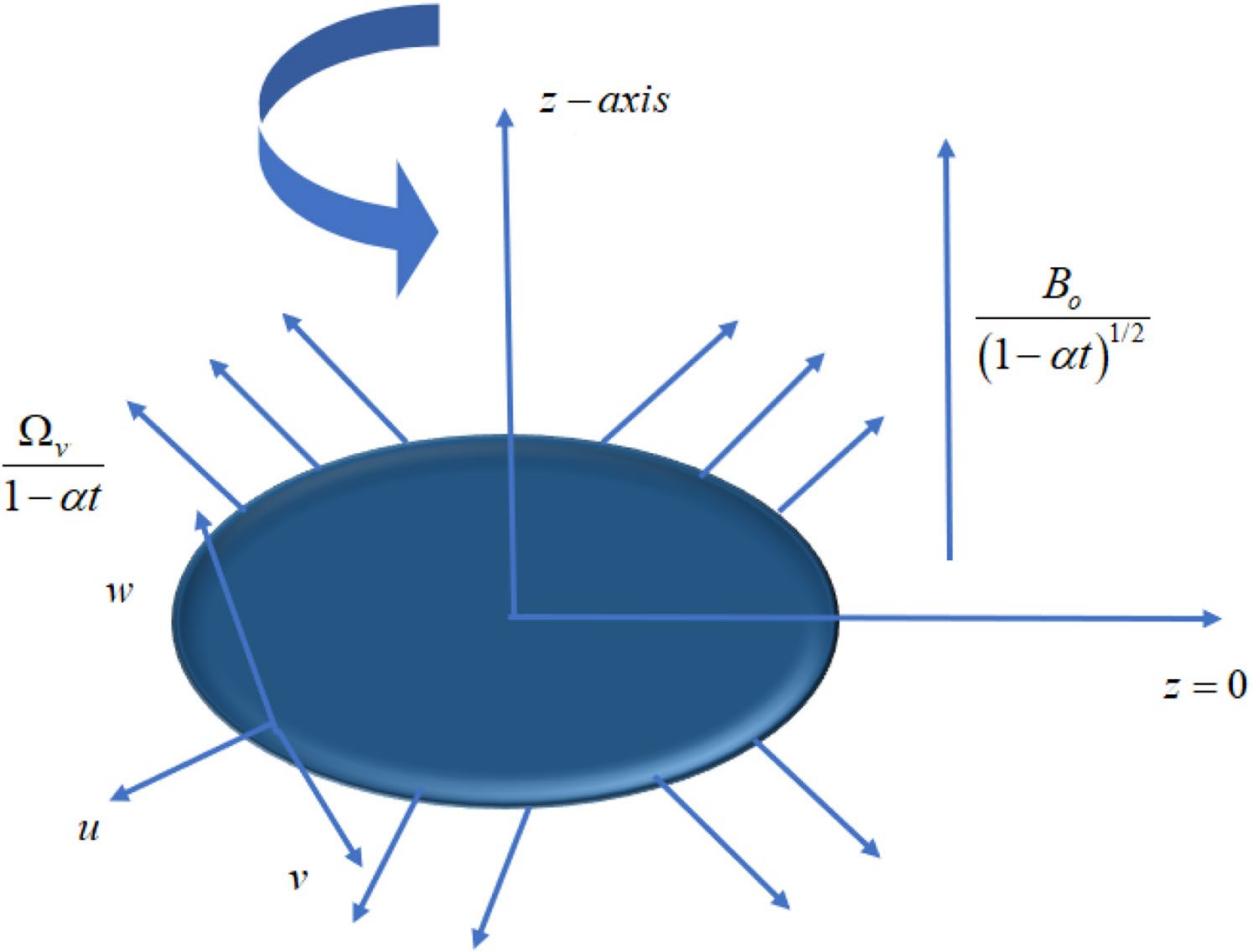
Geometrical sketch of the flow pattern.

The axially symmetric, non-conducting, incompressible nano-ferrofluid flow with velocity $\vec{V}^{*}$ past a rotating stretchable disk with applied magnetic field $\vec{\tilde{B}}^{*}$ with a magnitude $B_{o}$ as shown in Fig. 1. The disk is stretching with a stretching rate $\frac{{\Omega_{v} r}}{1 - \alpha t}$. Angular velocity of the disk is $\frac{{\alpha_{1} \Omega_{v} r}}{1 - \alpha t}$. The magnetization of the fluid is represented by the vector $\vec{\tilde{M}}^{*}$ with the strength of the magnetic field is $\vec{\tilde{H}}^{*}$. The basic continuity, momentum, magnetization, and rotational equations are given as^20^:

1 $$ \vec{\nabla } \cdot \vec{V}^{*} = 0, $$

2 $$ \rho_{nf} \frac{{d\vec{V}^{*} }}{dt} = - \nabla \vec{p} + \mu_{nf} \nabla^{2} \vec{V}^{*} + \mu_{0} (\vec{\tilde{M}}^{*} \cdot \nabla )\vec{\tilde{H}}^{*} + \frac{1}{2}\nabla \times (\vec{\tilde{M}}^{*} \times \vec{\tilde{H}}^{*} ) + \vec{\tilde{J}}^{*} \times \vec{\tilde{B}}^{*} . $$

The generalized form of Ohm law including electromagnetic effects are given as^18^:

3 $$ \vec{\tilde{J}}^{*} + \frac{{\omega_{e} t_{e} }}{{B_{o} }} \times \left( {\vec{\tilde{J}}^{*} \times \vec{\tilde{B}}^{*} } \right) - \sigma_{nf} (\vec{\tilde{E}} + \vec{V}^{*} \times \vec{\tilde{B}}^{*} ) - \frac{{\sigma_{nf} P_{e} }}{{en_{e} }} = 0. $$

With $\sigma_{nf}$ is the electrical conductivity of the ferrofluid. Assume that thermoelectric pressure is negligible.$n_{e}$ is the number density and $P_{e}$ is the pressure of electron. $\omega_{e}$ is the oscillating frequency of electrons. $t_{e}$ is the time for oscillatory frequency of electron. Where $\vec{\tilde{J}}^{*}$ represent the magnetic field having components^17,18^:

4 $$ J_{r} = \frac{{\sigma_{nf} \mu_{e} B_{o} }}{{1 + m^{2} }}\left( {u - mv} \right),J_{\varphi } = \frac{{\sigma_{nf} \mu_{e} B_{o} }}{{1 + m^{2} }}\left( {mu + v} \right). $$

The electric field is $\vec{\tilde{E}}^{*}$ which arises due to an electrically conducting magnetic field generated by charge separation. Where $\sigma_{nf} = \frac{{e^{2} n_{e} t_{e} }}{{m_{e} }}$ is the electrical conductivity of the fluid and $m = \omega_{e} t_{e}$ is the hall parameter. Assuming the molecular pressure and ion slip condition is negligible. Consider the electric and magnetic fields $\vec{\tilde{E}}^{*} (r,t) = \frac{{E_{o} r\Omega_{v} }}{{\left( {1 - \alpha t} \right)^{3/2} }},\vec{\tilde{B}}^{*} (t) = \frac{{B_{o} }}{{\left( {1 - \alpha t} \right)^{1/2} }}$.

The mean magnetic torque is given by Ellahi et al.^20^:

5 $$ \vec{\tilde{M}}^{*} \times \vec{\tilde{H}}^{*} = - 6\mu_{f} \phi \Omega g,g(\tilde{\xi }_{0} ,\tilde{\omega }_{0} \tilde{\tau }_{B} ) = \frac{1}{2}\tilde{\xi }_{0} \cos \tilde{\omega }_{0} \tilde{t}L^{*} (\tilde{\xi }_{e} )R^{*} (\tilde{\xi }_{e} ), $$

6 $$ \frac{1}{2}\nabla \times (\vec{\tilde{M}}^{*} \times \vec{\tilde{H}}^{*} ) = \frac{1}{2}\nabla \times - 6\mu_{f} \phi \Omega_{v} g = - \frac{3}{2}\mu_{f} \phi g\nabla (\nabla \cdot \vec{V}^{*} ) = \frac{3}{2}\mu_{f} \phi g\nabla^{2} \vec{V}^{*} . $$

By using Eq. (6) and (2) becomes:

7 $$ \rho_{nf} \frac{{d\vec{V}^{*} }}{dt} = - \nabla \vec{p} + \left( {\mu_{nf} + \frac{3}{2}\mu_{f} \phi g} \right)\nabla^{2} \vec{V}^{*} + \mu_{o} (\vec{\tilde{M}}^{*} \cdot \nabla )\vec{\tilde{H}}^{*} + \vec{\tilde{J}}^{*} \times \vec{\tilde{B}}^{*} . $$

Similarly, the temperature equation is given by^10^:

8 $$ \left( {\frac{{\partial \tilde{T}}}{\partial t} + \vec{V} \cdot \nabla \tilde{T}} \right) = k_{nf} (\nabla^{2} \tilde{T}). $$

So the continuity, momentum, and temperature equation gets the following form^17,18,20^:

9 $$ \frac{\partial u}{{\partial r}} + \frac{u}{r} + \frac{\partial w}{{\partial z}} = 0, $$

10 $$ \begin{aligned} \rho_{nf} \left( {\frac{\partial u}{{\partial t}} + u\frac{\partial u}{{\partial r}} + w\frac{\partial u}{{\partial z}} - \frac{{v^{2} }}{r}} \right) = & - \frac{{\partial p^{*} }}{\partial r} + \left( {\mu_{nf} + \frac{3}{2}\mu_{f} \phi g} \right) \\ \, \, & \times  \left( {\frac{{\partial^{2} u}}{{\partial r^{2} }} + \frac{1}{r}\frac{\partial u}{{\partial r}} - \frac{u}{{r^{2} }} + \frac{{\partial^{2} u}}{{\partial z^{2} }}} \right) + \frac{{\sigma_{nf} \vec{E}.\vec{B}}}{{1 + m^{2} }} - \frac{{\sigma_{nf} \vec{B}.\vec{B}}}{{1 + m^{2} }}\left( {u - mv} \right), \\ \end{aligned} $$

11 $$ \begin{aligned} \rho_{nf} \left( {\frac{\partial v}{{\partial t}} + u\frac{\partial v}{{\partial r}} + w\frac{\partial v}{{\partial z}} + \frac{uv}{r}} \right) = & \left( {\mu_{nf} + \frac{3}{2}\mu_{f} \phi g} \right) \\ \, & \times \left( {\frac{{\partial^{2} v}}{{\partial r^{2} }} + \frac{1}{r}\frac{\partial v}{{\partial r}} - \frac{v}{{r^{2} }} + \frac{{\partial^{2} v}}{{\partial z^{2} }}} \right) + \frac{{\sigma_{nf} \vec{E}.\vec{B}}}{{1 + m^{2} }} - \frac{{\sigma_{nf} \vec{B}.\vec{B}}}{{1 + m^{2} }}\left( {v + mu} \right), \\ \end{aligned} $$

12 $$ \rho_{nf} \left( {\frac{\partial w}{{\partial t}} + u\frac{\partial w}{{\partial r}} + w\frac{\partial w}{{\partial z}} - \frac{{v^{2} }}{r}} \right) = - \frac{{\partial p^{*} }}{\partial z} + \left( {\mu_{nf} + \frac{3}{2}\mu_{f} \phi g} \right)\left( {\frac{{\partial^{2} w}}{{\partial r^{2} }} + \frac{1}{r}\frac{\partial w}{{\partial r}} + \frac{{\partial^{2} w}}{{\partial z^{2} }}} \right), $$

13 $$ (\rho C_{p} )_{nf} \left( {\frac{{\partial \tilde{T}}}{\partial t} + u\frac{{\partial \tilde{T}}}{\partial r} + w\frac{{\partial \tilde{T}}}{\partial z}} \right) = k_{nf} \left( {\frac{{\partial^{2} \tilde{T}}}{{\partial r^{2} }} + \frac{1}{r}\frac{{\partial \tilde{T}}}{\partial r} + \frac{{\partial^{2} \tilde{T}}}{{\partial z^{2} }}} \right). $$

With boundary conditions are given by:

14 $$ \begin{gathered} u = \frac{{\alpha_{1} \Omega_{v} r}}{1 - \alpha t},v = \frac{{\Omega_{v} r}}{1 - \alpha t},w = 0, - k_{f} \frac{{\partial \tilde{T}}}{\partial z} = h_{f} (T_{f} - \tilde{T}),\quad {\text{at}}\,z = 0 \hfill \\ u = 0,v = 0,w = 0,\tilde{T}(r,\theta ,z) = T_{\infty } . \hfill \\ \end{gathered} $$

The mathematical form of thermophysical properties of nanofluid is given by^20^:

15 $$ A = \frac{{\mu_{nf} }}{{\mu_{f} }} = \frac{1}{{\left( {1 - \frac{{\phi_{a} }}{{\phi_{\max } }}} \right)^{{2.5\phi_{\max } }} }}, $$

16 $$ B = \frac{{\rho_{nf} }}{{\rho_{f} }} = \left( {1 - \phi_{a} } \right) + \frac{{\rho_{a} }}{{\rho_{f} }}\phi_{a} , $$

17 $$ C = \frac{{(\rho C_{p} )_{nf} }}{{(\rho C_{p} )_{f} }} = (1 - \phi_{a} ) + \frac{{(\rho C_{p} )_{a} }}{{(\rho C_{p} ){}_{f}}}\phi_{a} , $$

18 $$ D = \frac{{k_{nf} }}{{k_{f} }} = \frac{{k_{a} + 2k_{f} + 2\phi_{a} (k_{a} - k_{f} )}}{{k_{a} + 2k_{f} - \phi_{a} (k_{a} - k_{f} )}}, $$

19 $$ A_{1} = \frac{{\sigma_{nf} }}{{\sigma_{f} }} = 1 + \frac{{3\phi_{a} \left( {\frac{{\sigma_{a} }}{{\sigma_{f} }} - 1} \right)}}{{\left( {\frac{{\sigma_{a} }}{{\sigma_{f} }} + 2} \right) - \left( {\frac{{\sigma_{a} }}{{\sigma_{f} }} - 1} \right)}}. $$

The density and specific heat for aggregation of the particle are given as^20^:

20 $$ \frac{{\rho_{a} }}{{\rho_{f} }} = \left( {1 - \phi_{{\text{int}}} } \right) + \frac{{\rho_{s} }}{{\rho_{f} }}\phi_{{\text{int}}} , $$

21 $$ \frac{{(C_{p} )_{a} }}{{(C_{p} )_{f} }} = \left( {1 - \phi_{{\text{int}}} } \right) + \frac{{(C_{p} )_{s} }}{{(C_{p} )_{f} }}\phi_{{\text{int}}} , $$

22 $$ \phi_{{\text{int}}} = \phi_{c} + \phi_{nc} , $$

23 $$ \phi_{c} = \left( {\frac{{R_{g} }}{{a_{1} }}} \right)^{{d_{l} - 3}} ,\,\phi_{nc} = \left( {\frac{{R_{g} }}{{a_{1} }}} \right)^{{d_{f} - 3}} , $$

24 $$ N_{c} = \left( {\frac{{R_{g} }}{{a_{1} }}} \right)^{{d_{l} }} ,\,N_{{\text{int}}} = \left( {\frac{{R_{g} }}{{a_{1} }}} \right)^{{d_{f} }} . $$

Here, $N_{c}$ and $N_{{\text{int}}}$ are the number of particles in aggregation and belong to backbone particle. The contribution to thermal conduction is therefore given as^20^:

25 $$ (1 - \phi_{nc} )(k_{f} - k_{nc}^{*} )/(k_{f} + 2k_{nc}^{*} ) + \phi_{nc} (k_{s} - k_{nc}^{*} )/(k_{f} + 2k_{nc}^{*} ) = 0, $$

26 $$ k_{a}^{*} = k_{nc}^{*} \frac{{3 + \phi_{c} \left[ {2\alpha_{11}^{*} (1 - \beta_{11} ) + \alpha_{33}^{*} (1 - \beta_{33} )} \right]}}{{3 - \phi_{c} \left[ {2\alpha_{11}^{*} \beta_{11} + \alpha_{33}^{*} \beta_{33} } \right]}}, $$

27 $$\beta_{11}=0.5p^2/(p^2-1)-0.5p\text{Cosh}^{-1}p/(p^2-1)^{1.5},$$

28 $$ \beta_{33} = 1 - 2\beta_{11} , $$

29 $$ \alpha_{ii}^{*} = (k_{ii}^{*c} - k_{nc}^{*} )/\left[ {k_{nc}^{*} - \beta_{ii} (k_{ii}^{*c} - k_{nc}^{*} )} \right]. $$

Interfacial resistance is given by:

30 $$ k_{ii}^{*c} = \frac{{k_{s} }}{{1 + \gamma \beta_{ii} \left( {\frac{{k_{s} }}{{k_{f} }}} \right)}},\gamma = \left( {2 + \frac{1}{p}} \right)\beta ,\,\beta = \frac{{A_{k}^{*} }}{{a_{1} }},p = R_{g} a_{1} . $$

$A_{k}^{*}$ is the Kapitza radius, $a_{1}$ is the radius of primary particles, and the average radius of gyration is $R_{g} .$ Using the following transformation into Eqs. (8)–(14)

31 $$ \begin{gathered} \eta = \sqrt {\frac{{\Omega_{v} }}{{\upsilon_{f} }}} \frac{z}{{\sqrt {1 - \alpha t} }},u(r,\theta ,z) = \frac{{\Omega_{v} r\tilde{F}(\eta )}}{1 - \alpha t},v(r,\theta ,z) = \frac{{\Omega_{v} r\tilde{G}(\eta )}}{1 - \alpha t}, \hfill \\ w = \sqrt {\frac{{\Omega_{v} \upsilon_{f} }}{1 - \alpha t}} \tilde{E}(\eta ),\tilde{\theta }(\eta ) = \frac{{\tilde{T} - T_{\infty } }}{{T_{f} - T_{\infty } }},\frac{{p^{*} }}{{\rho_{f} }} = \frac{{ - \Omega_{v} v_{f} }}{1 - \alpha t}\tilde{P}(\eta ). \hfill \\ \end{gathered} $$

To obtain the dimensionless form of $Ec$, we assume that $T_{f} = T_{\infty } + T_{o} r^{2}$. Where $T_{o}$ is a constant having dimension $\left[ {L^{ - 2} K} \right]$. The dimensionless nonlinear partial differential equation along with boundary condition takes the following form:

32 $$ 2\tilde{F}(\eta ) + \tilde{E}^{\prime}(\eta ) = 0, $$

33 $$ \begin{gathered} \frac{{\rho {}_{nf}}}{{\rho_{f} }}\left[ {\tilde{F}^{2} - \tilde{G}^{2} + \tilde{E}\tilde{F}^{\prime} + S\left( {\tilde{F} + \frac{\eta }{2}\tilde{F}^{\prime}} \right)} \right] = \hfill \\ \;\left( {\mu_{nf} + \frac{3}{2}\mu_{f} \phi g} \right)\tilde{F}^{\prime\prime} - A_{1} M\left[ {\frac{1}{{1 + m^{2} }}\left( {\tilde{F} - m\tilde{G}} \right) - E_{1} } \right], \hfill \\ \end{gathered} $$

34 $$ \frac{{\rho_{nf} }}{{\rho_{f} }}\left[ {\tilde{E}\tilde{G}^{\prime} + 2\tilde{F}\tilde{G} + S\left( {\tilde{G} + \frac{\eta }{2}\tilde{G}^{\prime}} \right)} \right] = \left( {\mu_{nf} + \frac{3}{2}\mu_{f} \phi g} \right)\tilde{G}^{\prime\prime} - A_{1} M\left[ {\frac{1}{{1 + m^{2} }}\left( {\tilde{G} + m\tilde{F}} \right) - E_{1} } \right], $$

35 $$ \frac{{\rho_{nf} }}{{\rho_{f} }}\left[ {\tilde{E}\tilde{E}^{\prime} + \frac{S}{2}\left( {\tilde{E} + \frac{\eta }{2}\tilde{E}^{\prime}} \right)} \right] = \left( {\mu_{nf} + \frac{3}{2}\mu_{f} \phi g} \right)\tilde{E}^{\prime\prime}, $$

36 $$ C\left( {\frac{S\eta }{2} + \tilde{E}} \right)\tilde{\theta }^{\prime} + MEc\Pr \left( {E_{1}^{2} - 2E_{1} f^{\prime} + f^{^{\prime}2} } \right) = \frac{D}{\Pr }\tilde{\theta }^{\prime\prime}, $$

37 $$ \tilde{F}(0) = \alpha ,\tilde{F}(\infty ) = 0,\tilde{G}(0) = 1,\tilde{G}(\infty ) = 0,\tilde{E}(0) = 0,\tilde{\theta }^{\prime}(0) = Bi(\tilde{\theta }(0) - 1),\tilde{\theta }(\infty ) = 0, $$

where

38 $$ Bi = \frac{{h_{f} }}{{k_{f} }}\sqrt {\frac{{\upsilon_{f} (1 - \alpha t)}}{{\Omega_{v} }}} ,\,M = \frac{{\sigma_{f} B_{o}^{2} \left( {1 - \alpha t} \right)}}{{\rho_{f} \left( {1 + m^{2} } \right)}},\,\Pr = \frac{{\upsilon_{f} (\rho C_{p} )_{f} }}{{k_{f} }},E_{1} = \frac{{E_{o} }}{{B_{o} }},\,Ec = \frac{{\Omega_{v}^{2} }}{{C_{p} T_{o} }},\,S = \frac{\alpha }{{\Omega_{v} }}. $$

### Surface drag force and heat transfer rate

Mathematically drag force and rate of heat transfer is:

39 $$ C_{f} = \frac{{\sqrt {\tau_{wr}^{2} + \tau_{w\phi }^{2} } }}{{\rho_{f} \left( {\frac{{\alpha_{1} \Omega_{v} r}}{1 - \alpha t}} \right)^{2} }},Nu = \frac{{rq_{w} }}{{k_{f} (T_{f} - T_{\infty } )}}, $$

where $\tau_{wr}$ and $\tau_{w\phi }$ are the shear stresses in the radial and transverse directions respectively, which are given by.

40 $$ \tau_{wr} = \mu_{f} \left( {A + \frac{3}{2}\phi g} \right)\left( {\frac{\partial u}{{\partial z}} + \frac{\partial w}{{\partial \theta }}} \right)_{\begin{subarray}{l} z = 0 \\ q_{w} = - k_{nf} (T_{z} )_{z = 0} \end{subarray} } ,\,\tau_{w\phi } = \mu_{f} \left( {A + \frac{3}{2}\phi g} \right)\left( {\frac{\partial v}{{\partial z}} + \frac{1}{r}\frac{\partial w}{{\partial \theta }}} \right)_{z = 0} . $$

Inserting Eq. (38) into Eq. (37), we obtain

41 $$ Re^{1/2} C_{f} \left( {\mu_{nf} + \frac{3}{2}\mu_{f} \phi g} \right)\left( {\tilde{F}^{\prime}(0)^{2} + \tilde{G}^{\prime}(0)^{2} } \right)^{1/2} ,{\text{Re}}^{ - 1/2} Nu = \frac{{ - k_{nf} }}{{k_{f} }}\tilde{\theta }^{\prime}(0). $$

## Entropy generation analysis

Entropy is a thermophysical property that describes the rate of disorder or a measure of the chaos of a system and is given by

42 $$ \begin{aligned} S_{G} = & \frac{{k_{nf} }}{{T_{w} }}\left[ {\frac{{\partial \tilde{T}}}{\partial z}} \right]^{2} + \frac{{\mu_{nf} + \frac{3}{2}\mu_{f} \phi g}}{{T_{w} }} \\ & \times 2\left[ {\left( {\frac{\partial u}{{\partial r}}} \right)^{2} + \frac{{u^{2} }}{{r^{2} }} + \left( {\frac{\partial w}{{\partial z}}} \right)^{2} } \right] + \left( {\frac{\partial v}{{\partial z}}} \right)^{2} + \left( {\frac{\partial u}{{\partial z}}} \right)^{2} + \left[ {r\frac{\partial }{\partial r}\left( \frac{v}{r} \right)} \right]^{2} + \frac{{\sigma_{nf} B_{0}^{2} }}{{T_{w} }}(u^{2} + v^{2} ). \\ \end{aligned} $$

The entropy generation number is obtained by applying the transformation.

43 $$ \begin{gathered} \frac{{S_{G} }}{{S_{0} }} = N_{G} = D\varepsilon_{1} \tilde{\theta }^{{\prime}{2}} + \frac{{Br\left( {A + \frac{3}{2}\phi g} \right)}}{{\text{Re}}}[4\tilde{F}^{2} + 2\tilde{E}^{{\prime}{2}} + {\text{Re}} (\tilde{G}^{{\prime}{2}} + \tilde{F}^{{\prime}{2}} )] + A_{1} MBr(\tilde{F}^{2} + \tilde{G}^{2} ), \hfill \\ \hfill \\ \end{gathered} $$

where

44 $$ \, S_{0} = \frac{{k_{f} \Omega_{v} \Delta \tilde{T}}}{{\upsilon_{f} T_{f} (1 - \alpha t)}},Br = \frac{{\mu_{f} \Omega_{v}^{2} }}{{k_{f} (T_{f} - T_{\infty } )}},\varepsilon_{1} = \frac{\Delta T}{{T_{\infty } }},{\text{Re}}_{r} = \frac{{\Omega_{v} r^{2} }}{{\upsilon_{f} }}. $$

The Bejan number is the ratio of entropy created by thermal irreversibility to the total rate of disorder.

45 $$ Be = \frac{{\varepsilon_{1} \tilde{\theta }^{^{\prime}2} }}{{\varepsilon_{1} \tilde{\theta }^{^{\prime}2} + \frac{{Br\left( {A + \frac{3}{2}\phi g} \right)}}{{\text{Re}}}\left[ {4\tilde{F}^{2} + 2\tilde{E}^{^{\prime}2} + {\text{Re}} (\tilde{G}^{^{\prime}2} + \tilde{F}^{^{\prime}2} )} \right] + A_{1} MBr(\tilde{F}^{2} + \tilde{G}^{2} )}}. $$

## Analysis of results

**Figure 2:**
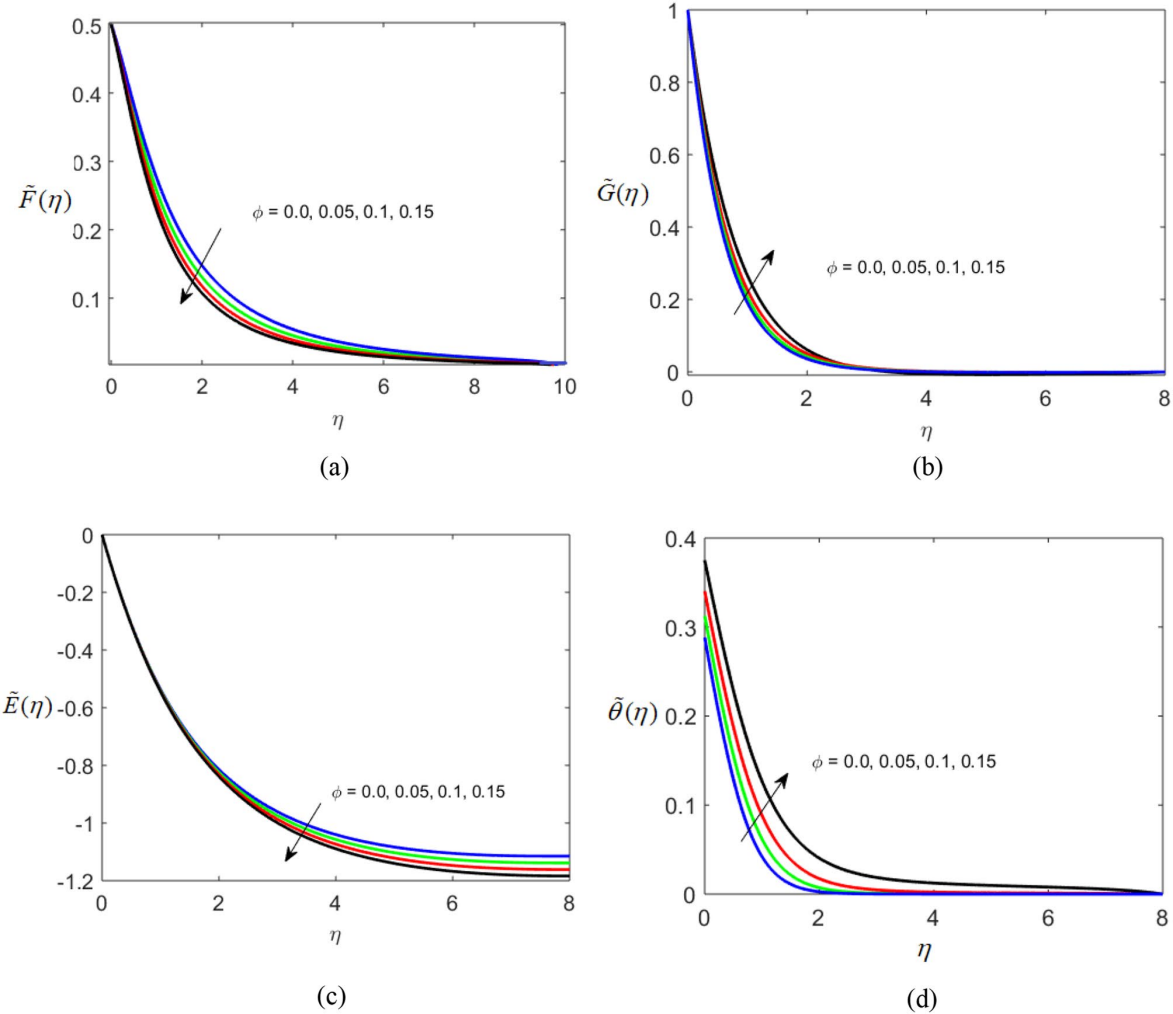
Radial, Tangential, axial profile of velocity, and thermal profile for increasing $\phi$.

**Figure 3:**
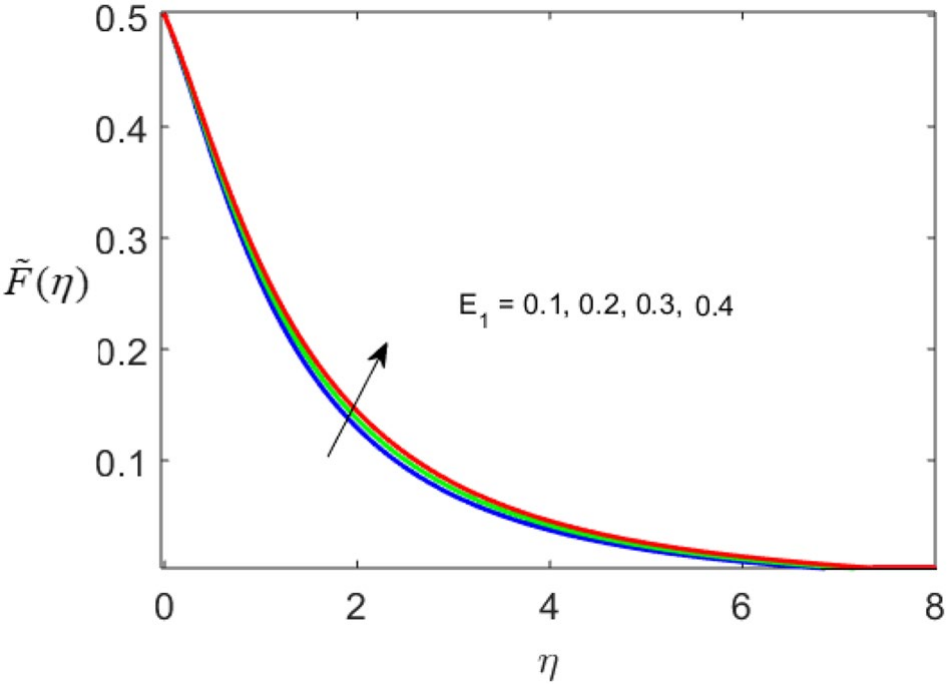
Variation of a radial velocity profile for electric parameter.

**Figure 4:**
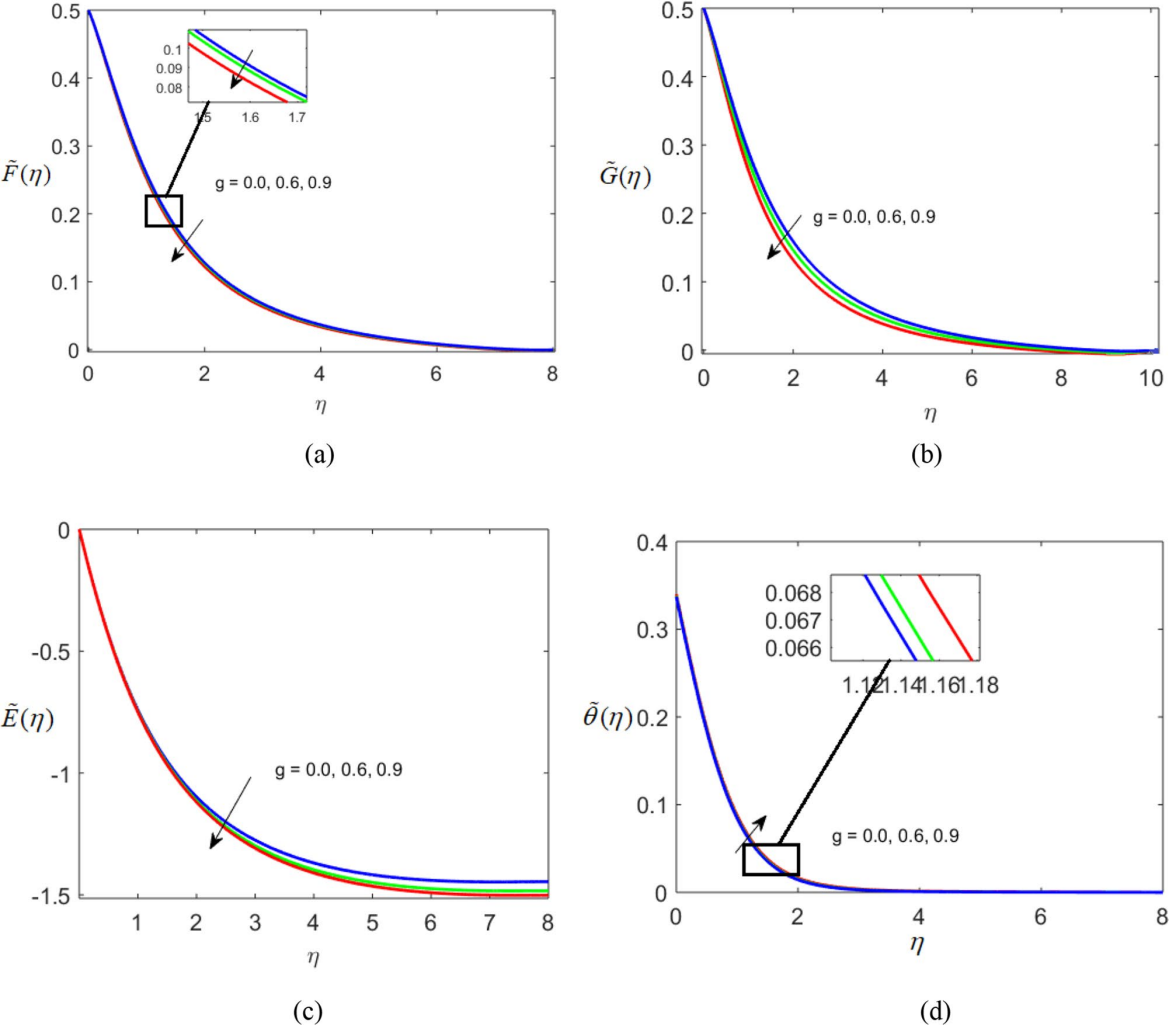
Radial, Tangential, axial profile of velocity, and thermal profile for increasing $g$.

**Figure 5:**
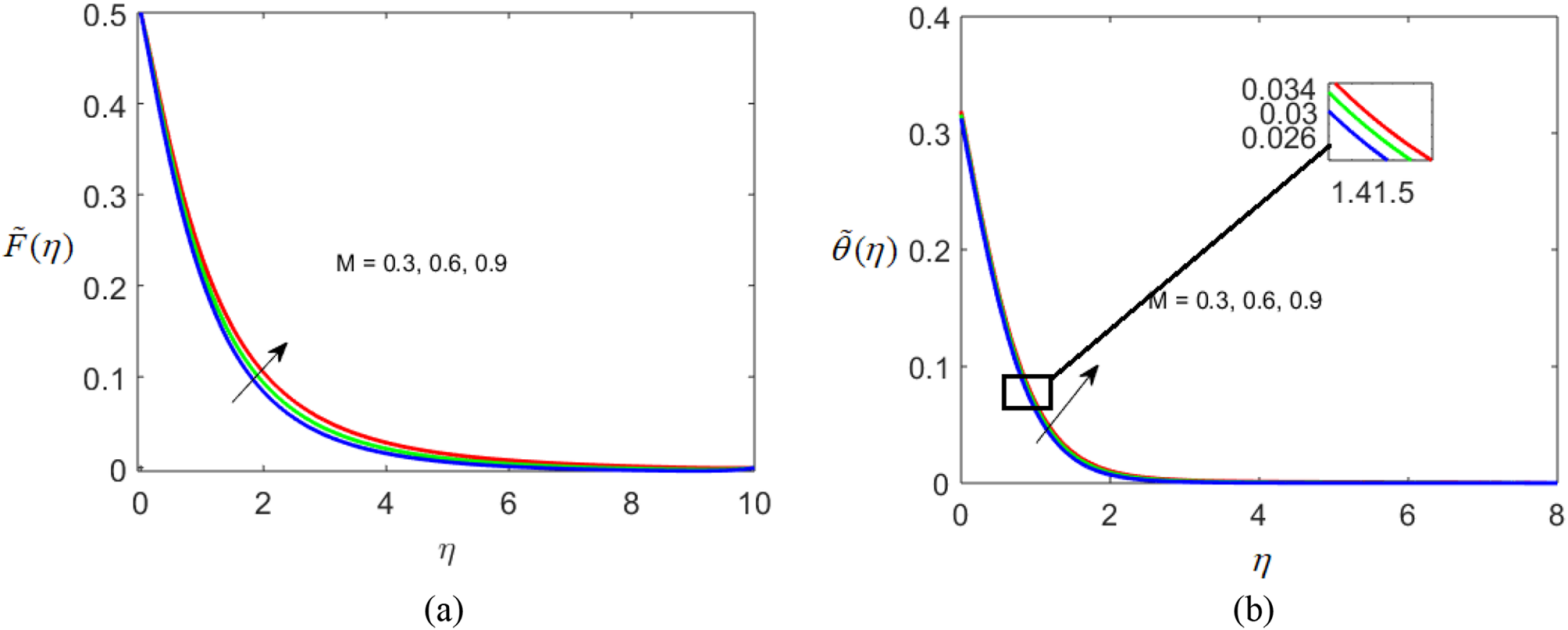
Radial profile of velocity and thermal profile for $M$.

**Figure 6:**
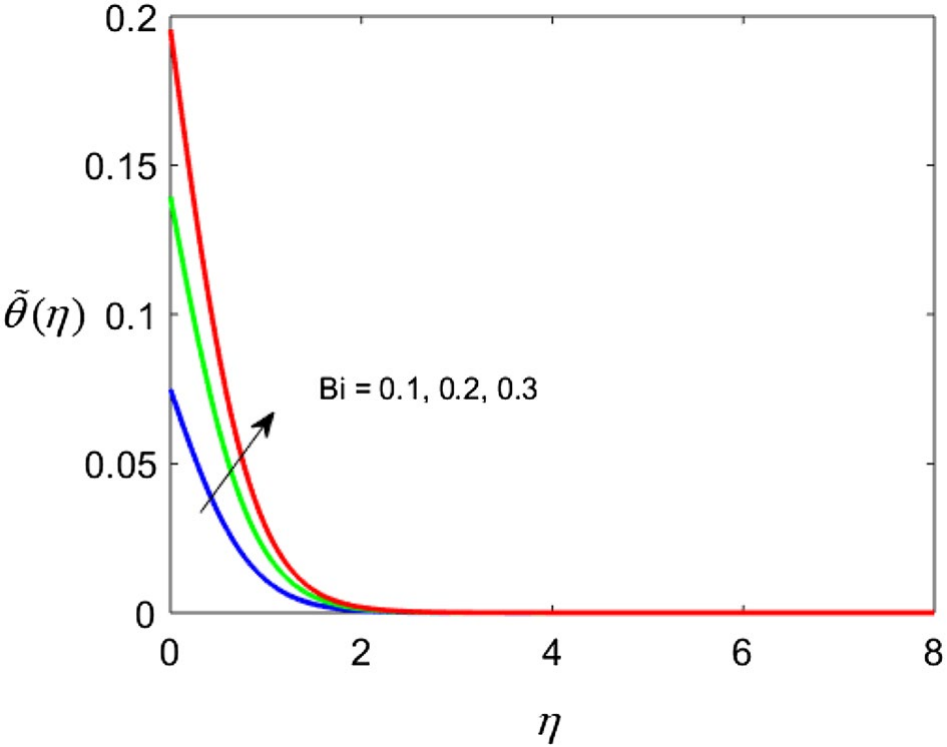
Thermal profile for $Bi$.

**Figure 7:**
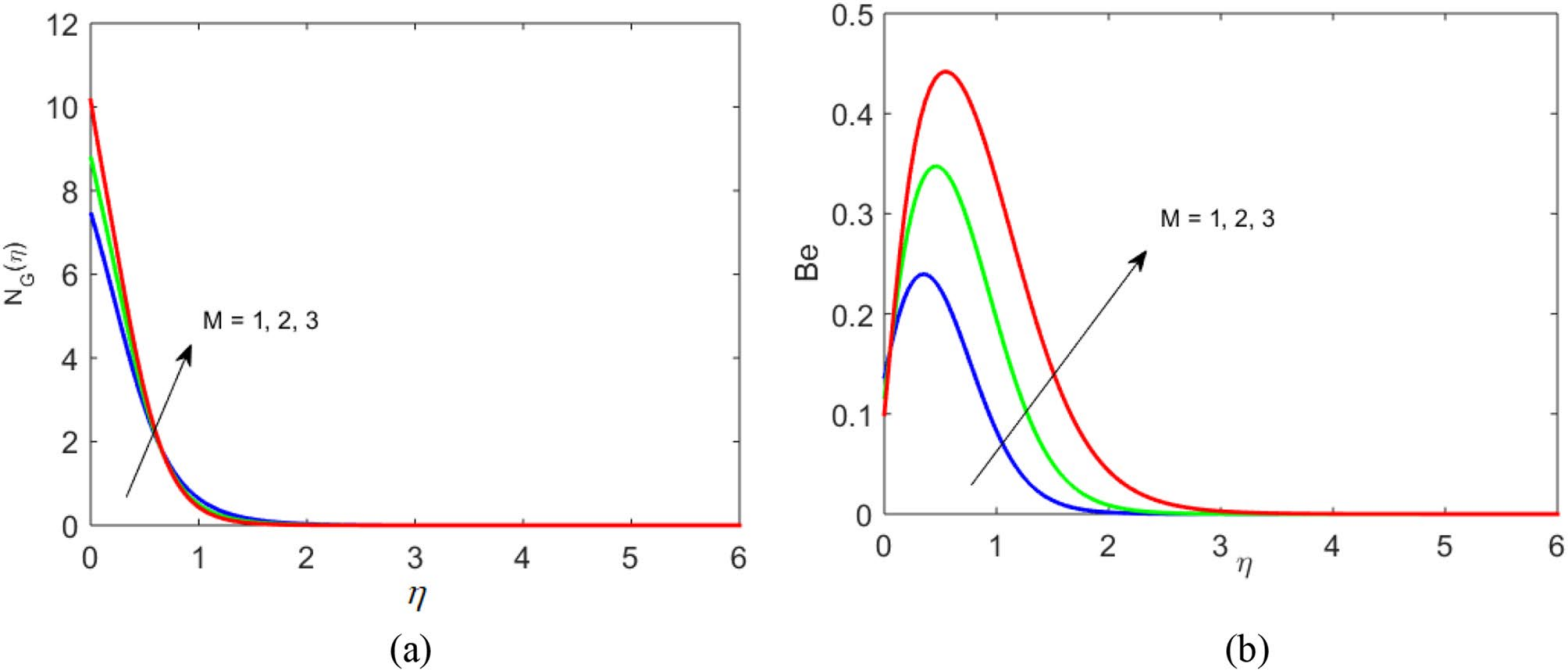
Profile of ${N}_{G}$ and $Be$ for $M$.

**Figure 8:**
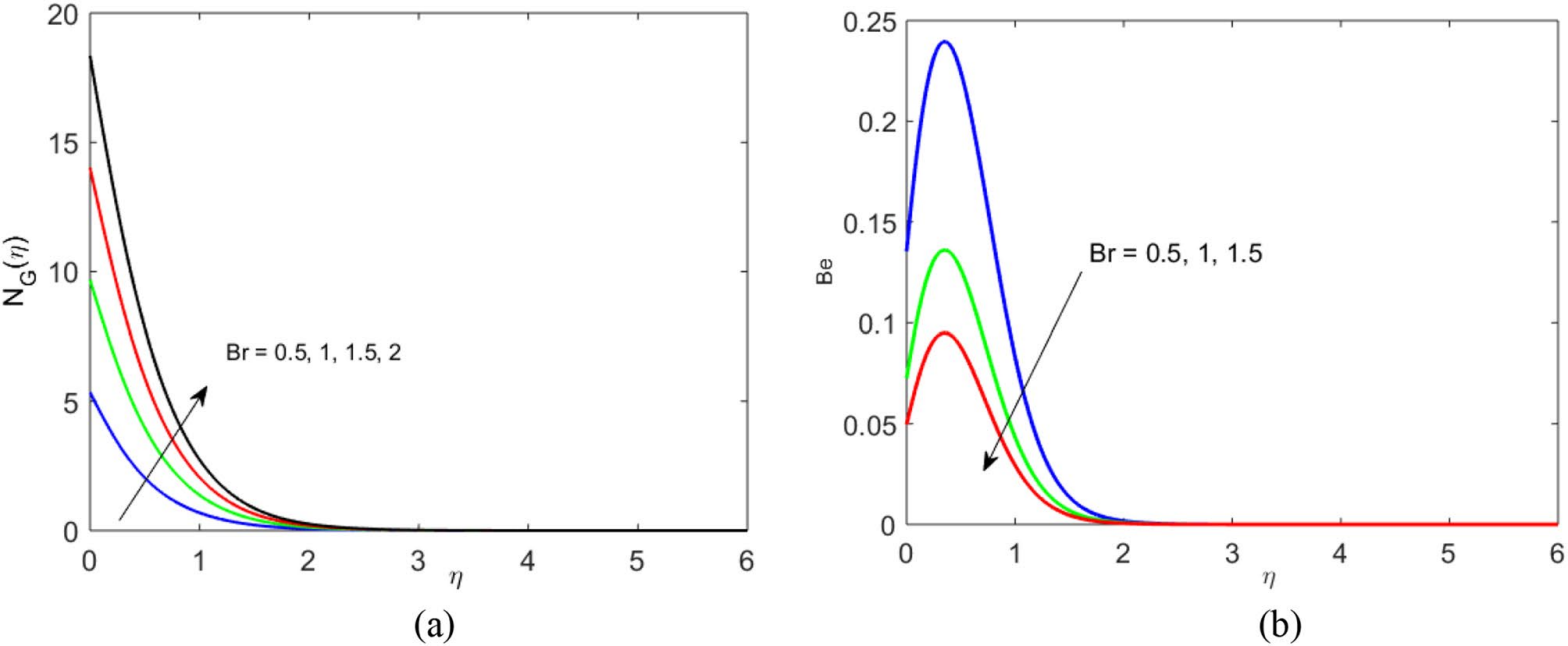
Profile of ${N}_{G}$ and $Be$ for $Br$.

**Figure 9:**
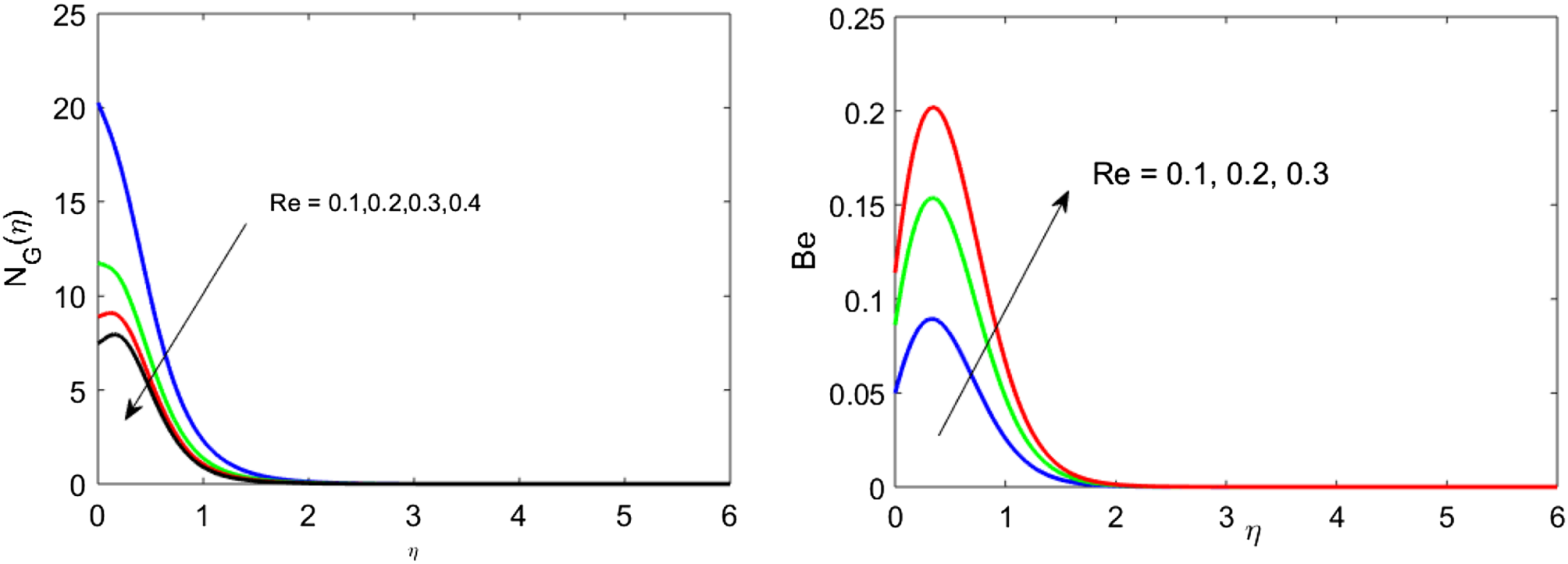
Profile of ${N}_{G}$ and $Be$ for $Re$.

The present section delineates the thermal and velocity profile through Figs. 2, 3, 4, 5, 6, 7, 8 and 9. We have considered the low oscillating magnetic field with Ferro liquid containing Iron as a nanoparticle. The nanoscale particles have a radius of 10 nm and the radius of gyration is 200 nm. Out of 200 particles in single aggregation, 50 particles are the backbone. To see the impact of nanoparticle volume fraction and effective magnetization parameter we fix the numerical value of $\alpha = 0.5$ and $S = 0.5$. The ranges of parameters are $0 \le \phi \le 0.15,0.1 \le E_{1} \le 0.4,0 \le g \le 0.9,0.3 \le M \le 3.0,0.1 \le Bi \le 0.3,0.5 \le Br \le 2,0.1 \le {\text{Re}} \le 0.4.$ Figure 2a–c represent radial, tangential, and axial velocity profiles by varying volume fraction. It is seen that radial and tangential velocity profiles are decreasing for escalating values of particle fraction this occurs because the resistive forces arise in the adjacent fluid layers. It shows that base liquid has more velocity than Ferro liquid. Figure 2d reveals that the cumulative nanoparticle concentration causes enhancement in the thermal field. The temperature of the Ferro liquid improves thermal conduction for increasing $\phi$. Figure 3 gives the graphical trend of radial velocity distribution for electric parameter $\left( {E{}_{1}} \right).$ The velocity boundary layer gets thicker by increasing the electric parameter. It causes the escalation of the radial velocity profile. Figure 4a,b depict the impact of effective magnetization parameters on the flow field. No significant enhancement in the behavior of radial and tangential velocity. As in the presence of a low oscillating magnetic field the ratio of the angular velocity of the particle to the angular velocity of the liquid declines which makes the magnetization parameter less dominant. Figure 4c depicts an opposite behavior. Figure 4d characterizes temperature profile for variation in $g$. The increasing thermal field behavior is due to enhancement in rotational viscosity. Figure 5a emphasizes the impact of $M$ on the radial velocity profile. Upon the escalating value of the Hall current parameter radial velocity increases. As the increasing value of the Hall parameter, the magnetic damping decreases along with propelling of the magnetic field causes the flow field to be enhanced. Figure 5b reveals that increasing Hartmann number causes temperature profile amplification. As the Lorentz force produces the resistance in particle’s motion which results in the enhancement in the thermal field. Figure 6 delineates the impact of the conjugate Newtonian heating parameter on the thermal field. The increasing temperature profile depicts that thermal boundary layer thickness is the function of the conjugate Newtonian heating parameter.

Figures 7, 8 and 9 portray the trend of rate of disorder and dimensionless pressure drop for various values of arising parameters such as $Br$, $Ec,$ and $Re$. Figure 7a,b show that upon the escalating values of the magnetic moment parameter, the entropy generation and Bejan number increase. Entropy is physically sensitive to magnetic moment parameters. As $M$ increases, the strength of interaction between fluid and applied magnetic field increases. The rotational viscosity decays, which causes the enhancement rate of chaos. Figure 8a,b represent the impact of the Brinkman number on the entropy generation and Bejan number. As the Brinkman number increase, it causes a rise in Joule heating, and heat generation intensifies. As a result, it causes an enhancement in the rate of disorder and $Be$ decrease. The impact of Reynolds number on ${N}_{G}$ and $Be$ is expressed in Fig. 9a,b. Increasing Reynolds number causes the enhancement of frictional forces in fluid flow to create the retarding force. And hence ${N}_{G}$ decrease. Moreover, heat transfer effects dominate over frictional forces causes an increase in Bejan’s number.

Table 1 enumerates surface drag force and heat transfer rate for varying the nanoparticle concentration and $g$. It is observed that an increase in $\phi$ and $g$ causes the enhancement in the velocity gradient and dynamic viscosity, which ultimately causes an increase in the drag force coefficient and heat transfer rate. Table 2 gives the comparison values of radial and tangential velocity at $\eta = 0.$

**Table 1:** Heat transfer rate and drag force for various values of $\phi$.

$g$	$\phi$(%)	$\sqrt {\text{Re}} C_{f}$	$\sqrt {\text{Re}} Nu$
0	1	1.69055	0.2576
0.6		1.75547	0.2611
0.9		1.78792	0.2628
1		1.79874	0.2634
0.1	0	1.35129	0.1976
	1	1.70137	0.2634
	5	2.11994	0.3484

**Table 2:** $\tilde{F}^{\prime}(0)$ and $\tilde{G}^{\prime}(0)$ for $\alpha = 2,M = 0,E_{1} ,m = 0.$

$S$	$\tilde{F}^{\prime}(0)$	$\tilde{F}^{\prime}(0)$	$\tilde{F}^{\prime}(0)$	$\tilde{G}^{\prime}(0)$	$\tilde{G}^{\prime}(0)$	$\tilde{G}^{\prime}(0)$
	Present	Rashidi^38^	Ellahi^20^	Present	Rashidi^34^	Ellahi^20^
− 0.1	− 3.1192	− 3.1178	− 3.1187	− 2.0529	− 2.0532	− 2.0530
− 0.5	− 2.9643	− 2.9601	− 2.9632	− 1.9900	− 1.9907	− 1.9901
− 1	− 2.7631	− 2.7622	− 2.7621	− 1.9102	− 1.9204	− 1.9111

## Concluding remarks

Unsteady nano ferrofluid flow for low oscillating magneto-viscous flow over a rotating stretchable disk has been explored in the present study. Velocity and thermal profiles are analyzed graphically for various values of arising parameters. Entropy generation rate is also evaluated for magnetic moment parameter, Brinkman number, Eckert number, and Reynolds number. The main findings of our observations are

- For increasing nanoparticle volume fraction and effective magnetization parameter radial and tangential velocity decrease while thermal profile increases.
- The radial velocity profile is an increasing function of electric and magnetic moment parameters.
- For increasing values Reynolds number and magnetic moment parameter, $Be$ amplifies and declines for $Br$.

## Nomenclature

$$r$$: Radial coordinate
$$\theta$$: Tangential coordinate
$$z$$: *z-*Coordinate
$$\vec{V}^{*} = (u,v,w)$$: Velocity
$$\Omega_{v}$$: Angular velocity
$$\alpha$$: Dimensional constant
$$\vec{p}^{*}$$: Pressure
$\rho_{nf}$: The density of the nanofluid
$\rho_{s}$: The density of solid single particles
$\phi$: Total volume fraction
$\phi_{{\text{int}}}$: Volume fraction in a cluster
$\phi_{a}$: Volume fraction in aggregate
$\phi_{nc}$: The volume fraction of dead-end particles
$\phi_{c}$: The volume fraction of backbone particles
$g$: Effective magnetization parameter
$\tilde{\xi }_{0}$: The amplitude of the real magnetic field
$\tilde{\xi }_{e}$: The effective magnetic field parameter
$\tilde{\omega }_{0}$: Frequency of the real magnetic field
$\tilde{\tau }_{B}$: Brownian relaxation time
$$T_{\infty }$$: Ambient temperature
$(C_{p} )_{nf}$: Specific heat of nanofluid
$(C_{p} )_{s}$: Specific heat of solid single particles
$(C_{p} )_{f}$: Specific heat of the fluid
$(C_{p} )_{a}$: Specific heat of aggregate of particles
$\sigma_{nf}$: Electrical conductivity of nanofluid
$\sigma_{f}$: Electrical conductivity of the fluid
$\sigma_{a}$: Electrical conductivity of aggregate of particles
$\sigma_{s}$: Electrical conductivity of solid particles
$\rho_{nf}$: The density of the nanofluid
$\rho_{s}$: The density of the solid particles
$\rho_{a}$: The density for aggregation of the particle
$$Ec$$: Eckert number
$k_{nf}$: Thermal conductivity of the nanofluid
$k_{f}$: Thermal conductivity of the fluid
$k_{s}$: Thermal conductivity of the solid particles
$k_{a}^{*}$: Thermal conductivity of the particles in aggregate
$$\tilde{t}$$: Time coordinate
$L^{*} (\tilde{\xi }_{e} )$: Effective Langevin function
$\vec{\tilde{J}}^{*}$: Magnetic field density
$$\vec{\tilde{B}}^{*}$$: Magnetic field
$B_{o}$: The magnitude of the magnetic field
$\tilde{T}$: The temperature of the fluid
${{\varvec{\Omega}}}$: The vorticity of the flow
$\vec{\tilde{E}}$: Induced electric field
$$\beta$$: Hall’s factor
$\vec{\tilde{M}}^{*}$: Magnetization of the fluid
$\vec{\tilde{H}}^{*}$: Magnetic field strength
$n$: Electron’s concentration per unit volume
$$q$$: Electronic charge
$T_{f}$: Reference temperature of the fluid
$Bi$: Local Biot number
$$M$$: Hartman number
$$\Pr$$: Prandtl number
$\mu_{nf}$: The viscosity of the nanofluid
$\mu_{0}$: Permeability of the free space
$\mu_{f}$: The viscosity of the fluid
${\text{Re}}_{r}$: Local Reynolds number
$$m$$: Hall’s parameter
$$Br$$: Brinkman number
$\varepsilon_{1}$: Non-dimensional temperature difference
$\rho_{a}$: The density for aggregation of the particle
$\rho_{s}$: The density of the solid particles
$$E{}_{1}$$: Electric parameter
$$S$$: Unsteadiness parameter
